# The Two Knowledges

**Published:** 1890-09-13

**Authors:** 


### The Hospital

A Weekly Journal of

Science, Medicine, Nursing, and Philanthropy.

*For Hospitals, Asylums, Medical Practitioners, Students, Nurses, Families, Parishes, Congregations, and the Charitable Public.*

Vol. VIII.—No. 207.] September 13, 1890.

### THE TWO KNOWLEDGES.

No man can learn to be a skilful doctor in four years. If any Admirable Crichton of a medical student were to take the first gold medal in every class during the whole of his curriculum he would still be a tyro at the bedside. Very few student readers will believe this; but every honest and capable man will find out its truth for himself before he has been ten years in practice. Is there a person anywhere, over twenty years of age, who has not discovered that there are two kinds of knowledge, and that they differ profoundly one from another? The knowledge obtained from the reading of books, the hearing of lectures, and from conversation is one thing; the knowledge acquired by personal and responsible handling of the things we profess to know about is something altogether different. The former is second-hand knowledge, and it is taken on trust; the latter is first hand, and it is personal property, acquired and proved by personal experience.

There is no intention here to depreciate second-hand knowledge. It is, indeed, the ladder by which practically every man must climb to personal experience and knowledge at first hand. What is dangerous, and to be constantly protested against, is that young men almost universally make the mistake of regarding their second-hand information as equal, or even superior, to the first-hand experience of other men. What a very great personage, for example, is the house physician who is known to have two or three medals hidden away somewhere among his belongings! The third-year's student looks upon him with awe; whilst he himself regards the awe as the most natural thing in the world; and sometimes wonders that it is not even greater than it is. If anybody were to tell him that many a country practitioner, who has forgotten his anatomy, and remembers his physiology no more, is as much superior to him in all the practical duties of a physician as he himself is better than the third-year's student he would stand amazed. "The country doctor," he would exclaim, "would be plucked in an examination even in practical medicine and surgery; how, then, can he be a more competent person at the bedside than the house physician who could pass with distinction in those very subjects?" Neither the house physician nor the senior student believes for a moment in the superior capacity of the country doctor. On the contrary, they commonly regard him with very sincere contempt.

There are books published on the art of whist-playing; and there is no difficulty in imagining that a clever young fellow, who had never played a game in his life, might read one or more of such books so thoroughly as to be able to take high honours in the theory and practice of whist. But it is quite as easy to imagine, and even to be sure, that such a man might be beaten in every game by an experienced player, who had never read a book on whist in his life, and who would infallibly be plucked at a whist examination. Do we, then, say that no knowledge is of any value, except such as is gained by practice and absolutely at first hand? Nothing could be further from the truth! But if a man is ever to become a really capable physician or surgeon, he must sooner or later learn the distinction between first and secondhand knowledge; and if he learns it sooner rather than later the better will it be for him. Whatever be the case with other professions, it is certain that vast numbers of medical men never acquire any true and proper first-hand knowledge at all. Even among those who practise in the higher ranks of the consultants there are considerable numbers who never had an original thought in their lives on a medical subject. They lived on books as students, and their mental digestion then became so accustomed to a diet of other men's ideas that it now refuses altogether to deal with anything that has not been cooked in another man's literary oven. Such practitioners, whether they be consultants or family physicians, are of the smallest possible use to their patients, and stand as a solid rock of obstruction in the pathway of true medical progress. It is amazing how much stupid medical literature they can take into their memories, and to what deeper depths of worthlessness they can degrade that which was worthless from the first. The consultant or the family doctor who is nothing but a reader is like one of those fat Hindoos whose spleen is large and rotten; a single kick is all that is necessary to put the finishing stroke to his pretentious and futile activities. A well-trained and experienced hospital nurse is worth a whole army of such physicians.

Medical education in a surprising number of instances is a complete failure. It is not, in fact, an education at all, but a stuffing, such as chickens undergo when they are stuffed by machinery in order to be rapidly fattened for the market; and the results are the same—the chickens are ruined as chickens by such a process, even for the table, and so are students. Medical professors will in most cases urge upon men the necessity of committing to memory and sticking to certain established methods of practice. They would do no harm if they would explain that such methods are only for temporary use until each man shall have acquired the capacity to originate methods of his own. He may even then choose to follow some particular professor's practice occasionally or perhaps frequently; but experience will have made that practice as much a part of himself as it originally was of the professor from whom it was learnt.

The thing to be insisted upon is that the medical student is learning how to think medically as well as how to practice an art. The third-rate student devotes himself exclusively to learning the technique of his business; the capable man does not despise technique by any means; but he insists upon thinking. Whilst, therefore, he commits steadily to memory, and "crams" for his examinations, he is always conscious that he is accepting statements on trust, and putting them away on the shelves of his memory for the present, with the fixed intention of taking them down again and turning them inside out at the first opportunity. The worst of all students is the mechanical grinder; and the miserablest of all grinders is the medical. What an insufferable prig he is; and how useless at the bedside! He can reel off theories by the mile; but he never pretends to decide upon their value. To him a theory is a theory, and a name is a name; and if he happens to know one more theory or one more name than any of his competitors he is abundantly content. Of course he takes honours; and, of course, he gets hospital appointments; and equally, of course, he makes the educated members of other professions stare with astonishment at the strength of his memory, and at the feebleness of his thought. Perhaps there is more of slavery to books and names, and less of mental originality in the medical profession than in any other. This is partly the fault of the powerful traditions which still cling to the average medical mind. But it is probably still more largely due to the "cram" system. Medical examinations have now reached a maximum of absurdity both in their extent and in their unpracticality. Perhaps some of the more capable examiners will take the matter to heart, and have the courage to think out and advocate a better way. But in any case every medical student who values his own mind and character should resolve that at all risks he will preserve his power of independent and original thought, whether he crams successfully or not.

